# Supplementary figures and images for: Mutations in the SmAPRR2 transcription factor suppressing chlorophyll pigmentation in the eggplant fruit peel are key drivers of a diversified colour palette
Source: Front Plant Sci. 2022 Oct 27;13:1025951. doi: 10.3389/fpls.2022.1025951 (PMC9647125; doi:10.3389/fpls.2022.1025951)

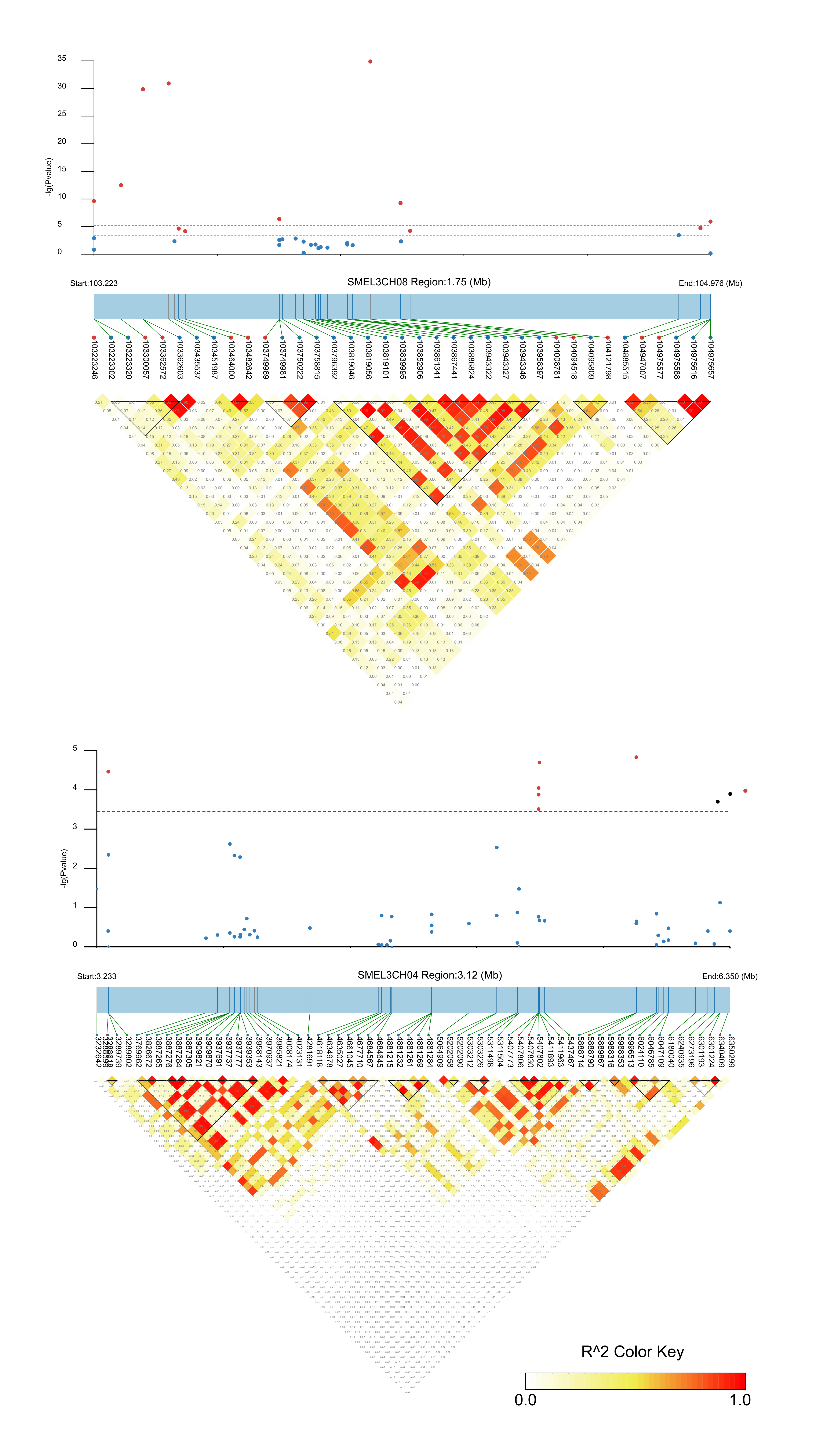

Supplement: Supplementary Figure 1 — Local Manhattan plot and LD heatmap of the candidate peaks on chromosomes 8 and 4, top and bottom figures, respectively. The red and green horizontal lines represent, respectively, FDR and Bonferroni significance thresholds at p=0.05. In red are represented the points that exceed these thresholds, while in blue are the points that do not. Pairwise LD between SNPs is indicated as r2 values: red indicates a value of 1 and white indicates 0. [file Image_1.png]
